# Comparison and Sensitivity Evaluation of Three Different Commercial Real-Time Quantitative PCR Kits for SARS-CoV-2 Detection

**DOI:** 10.3390/v13071321

**Published:** 2021-07-08

**Authors:** Ana Banko, Gordana Petrovic, Danijela Miljanovic, Ana Loncar, Marija Vukcevic, Dragana Despot, Andja Cirkovic

**Affiliations:** 1 Institute of Microbiology and Immunology, Faculty of Medicine, University of Belgrade, 11000 Belgrade, Serbia; danijela.karalic@med.bg.ac.rs; 2 Institute for Biocides and Medical Ecology, 11030 Belgrade, Serbia; goca_petrovic@yahoo.com (G.P.); dranaloncar@gmail.com (A.L.); marija.vukcevic@biocidi.ord.rs (M.V.); drdespot@hotmail.com (D.D.); 3 Institute for Medical Statistics and Informatics, Faculty of Medicine, University of Belgrade, 11000 Belgrade, Serbia; andja.aleksic@gmail.com

**Keywords:** SARS-CoV-2, COVID-19, real-time PCR, molecular testing, PCR kits, diagnostic efficacy

## Abstract

Real-time reverse transcription polymerase chain reaction (RT-qPCR) is the most sensitive and specific assay and, therefore, is the “gold standard” diagnostic method for the diagnosis of SARS-CoV-2 infection. The aim of this study was to compare and analyze the detection performance of three different commercially available SARS-CoV-2 nucleic acid detection kits: Sansure Biotech, GeneFinder^TM^, and TaqPath^TM^ on 354 randomly selected samples from hospitalized COVID-19 patients. All PCR reactions were performed using the same RNA isolates and one real-time PCR machine. The final result of the three evaluated kits was not statistically different (*p* = 0.107), and also had a strong positive association and high Cohen’s κ coefficient. In contrast, the average Ct values that refer to the ORF1ab and N gene amplification were significantly different (*p* < 0.001 and *p* < 0.001, respectively), with the lowest obtained by the TaqPath^TM^ for the ORF1ab and by the Sansure Biotech for the N gene. The results show a high similarity in the analytical sensitivities for SARS-CoV-2 detection, which indicates that the diagnostic accuracy of the three assays is comparable. However, the SanSure Biotech kit showed a bit better diagnostic performance. Our findings suggest that the imperative for improvement should address the determination of cut-off Ct values and rapid modification of the primer sets along with the appearance of new variants.

## 1. Introduction

From the first case of coronavirus disease 2019 (COVID-19) in December 2019 until mid-April 2021, there were more than 147 million confirmed cases and 3.1 million deaths. According to exponential growth in the number of infections globally, COVID-19 has become one of the greatest pandemics in modern history. It is caused by a novel virus, severe acute respiratory syndrome coronavirus 2 (SARS-CoV-2), which belongs to the family *Coronaviridae*, subfamily *Coronavirinae*, and genus *betacoronavirus*.

Coronaviruses are enveloped, positive-stranded RNA viruses with a genome nearly 29.9 kb in length. The genome consists of several genes that encode non-structural, structural, and accessory proteins [1]. The *ORF1a* and *ORF1b* genes encode two polyproteins that are cleaved into 16 non-structural proteins, such as RNA-dependent RNA polymerase (RdRp), helicase, and various proteases [2]. In the last third of the genome, genes for four structural proteins (spike surface glycoprotein S, envelope E, membrane M, and nucleocapsid N) and several accessory proteins are located [3]. The ORF1ab/RdRp, E, N, and S genes are the targets most frequently used for SARS-CoV-2 detection by real-time reverse transcription polymerase chain reaction (RT-qPCR) method [4]. In some set-ups, the E or RdRp gene primers are specific for bat (-related) betacoronaviruses, not only for SARS-CoV-2 [4].

The pandemic has created an enormous burden to public health systems, social circumstances and the global economy. Therefore, timely and accurate diagnosis of both symptomatic and asymptomatic human carriers of SARS-CoV-2 infection was identified as one of the key links in the successful management of COVID-19 spread [5]. RT-qPCR is the most sensitive and specific assay and, therefore, is the “gold standard” diagnostic method for the diagnosis of SARS-CoV-2 infection [6,7]. 

Due to huge demands to increase the capacity of COVID-19 diagnostics, many laboratories for molecular detection of different infectious agents have shown readiness for the fast implementation of laboratory diagnostics of SARS-CoV-2. In that manner, testing has shifted away from specialized laboratories, which experienced a lack of staff, lab supplies, space and equipment, especially in the early phases of laboratory response. Moreover, challenges were identified in providing sufficient personnel with knowledge and experience in the aspect of clinical validation of specificity and sensitivity, where there is still room for improvement [7]. 

The first full-length SARS-CoV-2 genome sequence was made publicly available in January 2020, and soon after, various RT-qPCR assays, laboratory developed or commercially produced, were implemented in laboratory diagnosis [8]. According to the Foundation for Innovative New Diagnostics list [9], there are currently more than 430 commercialized/in development tests, but often without clear overall performance and relative sensitivity [10]. Whereas many COVID-19 RT-qPCR kits are currently being used, a continuous independent assessment of these products is necessary to perform in order to guide the implementation of accurate tests in the diagnostic market.

The aim of this study was to compare and analyze the detection performance of three different commercially available SARS-CoV-2 nucleic acid detection kits.

## 2. Materials and Methods

### 2.1. Specimen Collection

At the beginning of April 2020, the Laboratory of Molecular Microbiology, Institute for Biocides and Medical Ecology, Belgrade, was designated as one of the relevant laboratories for COVID-19 diagnosis and research. During March 2021, the local epidemic was at its fourth peak. From this period, 354 randomly selected specimens from hospitalized COVID-19 patients, originally submitted for routine SARS-CoV-2 diagnosis, were included in this study. One nasopharyngeal and one oropharyngeal swab were taken from each patient and placed into the same tube with 3 mL of viral transport medium (Liuyang SANLI Medical Technology). After the initial testing, samples were aliquoted and stored at −80 °C. 

### 2.2. Viral RNA Extraction

According to the manufacturer’s instructions, the viral RNA was extracted from the 200 µL of the samples using a Viral DNA/RNA Extraction Kit (ALPHAGENE Co. Ltd., Sengnam, Korea). Extraction was done on the automatic nucleic acid extraction system NC-15 plus (ALPHAGENE Co. Ltd.), also according to manufacture instructions. One elution volume per sample was enough to perform all RT-qPCR reactions from this study. This ensured that the RT-qPCR reactions being compared were taken from identical sample preparations. Eluates were stored at −80 °C. 

### 2.3. Molecular Assays

The three tests used in this study are the part of the routinely used tests in designated COVID-19 laboratories in the Republic of Serbia and the only ones currently used in the Laboratory of Molecular Microbiology, Institute for Biocides and Medical Ecology, Belgrade, where the mentioned experiments were performed. The three different molecular assays for the qualitative detection of SARS-CoV-2 that were used were: GeneFinder^TM^ COVID-19 Plus RealAmp Kit (OSANG Healthcare Co., Seongnam, Korea), Sansure Biotech (Sansure Biotech Inc., Changsha, China), and TaqPath^TM^ COVID-19 CE-IVD RT-qPCR Kit (Thermo Fisher Scientific, Waltham, MA, USA) according to manufacturer’s instructions. Specifications of these assays are shown in detail in Table 1. ORF1ab and N genes are detected by all three tests. Only the instructions for the GeneFinder^TM^ test specified the genomic position within the ORF1ab:RdRp gene; besides these two genes, additional gene targets are detected with GeneFinder^TM^ and TaqPath^TM^, E and S gene, respectively. To avoid the possible influence of machine performance on the outcome of the results, all PCR reactions were performed using the same real-time PCR machine (Quant Studio^TM^ 5 Real-Time PCR Instrument (Thermo Fisher Scientific, Waltham, MA, USA) under the following conditions: (i) for GeneFinder^TM^ COVID-19 Plus RealAmp Kit: reverse transcription at 50 °C for 20 min, followed by pre denaturation at 95 °C for 5 min, then 45 cycles at 95 °C for 15 s and 58 °C for 60 s for denaturation and annealing (collection of data); (ii) for Sansure Biotech: reverse transcription at 50 °C for 30 min, followed by denaturation at 95 °C for 1 min, then 45 cycles at 95 °C for 15 s and 60 °C for 31 s for denaturation and annealing (collection of data) and at the end cool down for the instrument at 25 °C for 10 s; (iii) for TaqPath^TM^ COVID-19 CE-IVD RT-qPCR Kit: UNG incubation at 25 °C for 2 min followed by reverse transcription at 53 °C for 10 min, then activation at 95 °C for 2 min, followed by 40 cycles at 95 °C for 3 s and 60 °C for 30 for denaturation and annealing/extension.

For quality control and quality assurance, negative, positive and internal controls for each assay were used. The controls for each run must meet assay requirements before a run is accepted as valid. The automation of threshold setting to 10,000 minimized variations in assay parameters that could affect the Ct values.

### 2.4. Analysis of the Results

The interpretation of test results was carried out following the manufacturer’s instructions for all three kits. For GeneFinder^TM^ COVID-19 Plus RealAmp Kit, a specimen is positive if a sigmoidal amplification curve in RdRp, N, and/or E gene with Ct values is not higher than 40. For GeneFinder^TM^, the result was considered positive if (1) only the ORF1ab gene was positive, (2) only N gene was positive (3) both ORF1ab and N gene were positive at the same time. If only an S-shape curve is detected in the E gene, it is called “a presumptive positive”, and the test must be repeated once from RT-qPCR. If the repeated result remains “presumptive positive” it is considered positive. For SunSure Biotech, a sigmoidal amplification curve in ORF1ab and/or N gene with Ct ≤ 40 represents a positive result. For this test, the RT-qPCR result is considered positive if: (1) only the ORF1ab gene was positive, (2) only the N gene was positive (3), or both ORF1ab and N gene were positive at the same time. For TaqPath^TM^ COVID-19 CE-IVD RT-qPCR Kit, an S-shape amplification curve in two or more target genes (ORF1ab, N, S) with Ct values ≤ 37 is interpreted as a positive result. When only one target gene is positive, retesting is needed. If the result remains the same, it is considered positive. For TaqPath^TM^, the result was considered positive in seven possible situations: (1) only the ORF1ab gene was positive, (2) only the N gene was positive, (3) only the S gene was positive, (4) both the ORF1ab and N genes were positive, (5) both the ORF1ab and S genes were positive, (6) both the N and S genes were positive, or (7) the ORF1ab and N and S genes were positive at the same time.

### 2.5. Statistical Analyses

Numerical data are presented as the arithmetic mean with standard deviation, because data had a normal distribution. The normality was evaluated according to mathematical methods (coefficient of variation, skewness and kurtosis, and Kolmogorov–Smirnov and Shapiro–Wilk tests) and graphical methods (histogram, box plot). Categorical variables are presented as absolute and relative numbers (percentages). One-way ANOVA with Tukey post hoc testing was used to compare numerical variables with normal distribution between three independent samples. The Chi-square test or its alternative Fisher’s exact test was applied for testing the difference in frequency of categorical variables within independent samples. To evaluate the reliability of three RT-qPCR methods, we used Pearson’s linear coefficient of correlation (r) and Cohen’s Kappa coefficient (κ). Altman’s scale of interpreting the strength of agreement was used for concluding [11]. In addition, diagnostic accuracy (sensitivity, Sn; specificity, Sp, overall accuracy, positive predictive value, PPV; negative predictive value, NPV; likelihood ratio for positive test, LR+; and likelihood ratio for negative test, LR−) were calculated by comparing all three methods among themselves. All statistical methods were considered significant for the level of confidence of 0.05. The analysis was done in statistical software IBM Corp. Released 2012. IBM SPSS Statistics for Windows, Version 21.0. Armonk, NY, USA: IBM Corp.

## 3. Results

A total of 354 randomly selected COVID-19 patients were enrolled in this cross-sectional study. Three different RT-qPCR methods were performed: Sansure Biotech, GeneFinder^TM^, and TaqPath^TM^. Results for all three methods are presented in Table 2.

The final result of the Sansure Biotech RT-qPCR method was significantly more often positive then the GeneFinder^TM^ and TaqPath^TM^ RT-qPCR methods (*p* < 0.001 and *p* < 0.001, respectively), and the GeneFinder^TM^ RT-qPCR had significantly more positive final results than the TaqPath^TM^ RT-qPCR method (*p* < 0.001).

Positive results of RT-qPCR methods, according to a single and multiple gene positivity, are also presented in Table 2. There was no statistically significant difference in the frequency of positive results of RT-qPCR according to the single ORF1ab gene positivity between all three methods (*p* = 0.059). There was no difference in the positive results of RT-qPCR method according to the single ORF1ab gene positivity between the SanSure Biotech and GeneFinder^TM^ (*p* = 0.406), but statistically significant difference was found between the SanSure Biotech and TaqPath^TM^ (*p* = 0.045), and GeneFinder^TM^ and TaqPath^TM^ method (*p* = 0.014). The frequency of positive results of all three evaluated RT-qPCR methods according to only N gene positivity, was statistically different (*p* = 0.030). Positive results according to only N gene was higher for the TaqPath^TM^ method in comparison to SanSure Biotech (*p* = 0.040), also it was significantly higher for the TaqPath^TM^ method than GeneFinder^TM^ (*p* = 0.024). On the other hand, no significant difference was observed between positive results for the SanSure Biotech and GeneFinder according to only N gene positivity (*p* = 0.832). When comparing all three evaluated RT-qPCR methods, a statistically significant difference in the frequency of positive results according to both ORF1ab and N gene positivity was shown (*p* < 0.001). The frequency of positive results according to both ORF1ab and N gene positivity was significantly higher for the Sansure Biotech method than TaqPath^TM^ (*p* < 0.001), as well as for the GeneFinder^TM^ than TaqPath^TM^ methods (*p* < 0.001). On the other hand, no significant difference in the frequency of positive RT-qPCR results, according to both the ORF1ab and N gene positivity, was found when comparing the SanSure Biotech and GeneFinder^TM^ (*p* = 0.292).

Ct values for all RT-qPCR methods and comparisons between them are shown in Table 3. The average Ct value was calculated as the arithmetic mean of all Ct values when the particular gene was considered positive (single or multiple gene RT-qPCR positivity). The average Ct values that refer to amplification of the ORF1ab gene obtained by TaqPath^TM^ RT-qPCR method were significantly lower than the Sansure Biotech and GeneFinder^TM^ (*p* < 0.001). TaqPath^TM^ Ct values that refer to amplification of the ORF1ab gene were significantly lower than the GeneFinder^TM^ Ct values (*p* < 0.001). In addition, the TaqPath^TM^ Ct values that refer to amplification of the ORF1ab gene were significantly lower than the Sansure Biotech Ct values (*p* = 0.008). The average Ct values that refer to amplification of N gene obtained by the Sansure Biotech RT-qPCR method were significantly lower than the values obtained by the GeneFinder^TM^ and TaqPath^TM^ (*p* < 0.001). Sansure Biotech Ct values were significantly lower than GeneFinder^TM^ Ct values, and TaqPath^TM^ Ct values were significantly lower than the GeneFinder^TM^ Ct values (*p* = 0.001). The Sansure Biotech and TaqPath^TM^ Ct values that refer to amplification of the ORF1ab gene did not differ (*p* = 0.171). In addition, there was no significant difference in Ct values that refer to amplification of N gene between the Sansure Biotech and TaqPath^TM^ methods (*p* = 0.797).

Table 4 shows the association between the Ct values obtained by three RT-qPCR methods. There was a strong positive significant association between the Ct values obtained by Sansure Biotech, GeneFinder^TM^, and TaqPath^TM^ RT-qPCR methods. This result suggested great reliability between methods.

Cohen’s d Kappa coefficient also confirmed the agreement between the Sansure Biotech, the GeneFinder^TM^ and the TaqPath^TM^ RT-qPCR methods. Comparison of the assessment of SARS-CoV-2 positivity according to the Sansure Biotech and the GeneFinder^TM^ produced κ = 0.914, which suggests a strong strength of agreement between the two methods (*p* < 0.001). In addition, a strong strength of agreement was shown between the Sansure Biotech and the TaqPath^TM^ methods with κ = 0.830, *p* < 0.001, and the GeneFinder^TM^ and TaqPath^TM^ methods with κ = 0.904 (*p* < 0.001).

Diagnostic performance for all three methods is presented in Table 5. Overall, all three RT-qPCR methods showed high overall diagnostic accuracy for detecting SARS-CoV-2 positive patients. In summary, the analysis of the tests showed a good sensitivity, while specificity was the highest for the Sansure Biotech compared to the GeneFinder^TM^ and the lowest for the Sansure Biotech in compared to the TaqPath^TM^. According to the highest sensitivity, the Sansure Biotech was the most accurate test. The likelihood ratio for a positive test (LR+) for the Sansure Biotech was higher than the GeneFinder^TM^, and the GeneFinder^TM^ had greater LR+ compared to the TaqPath^TM^ method.

**Figure 1:**
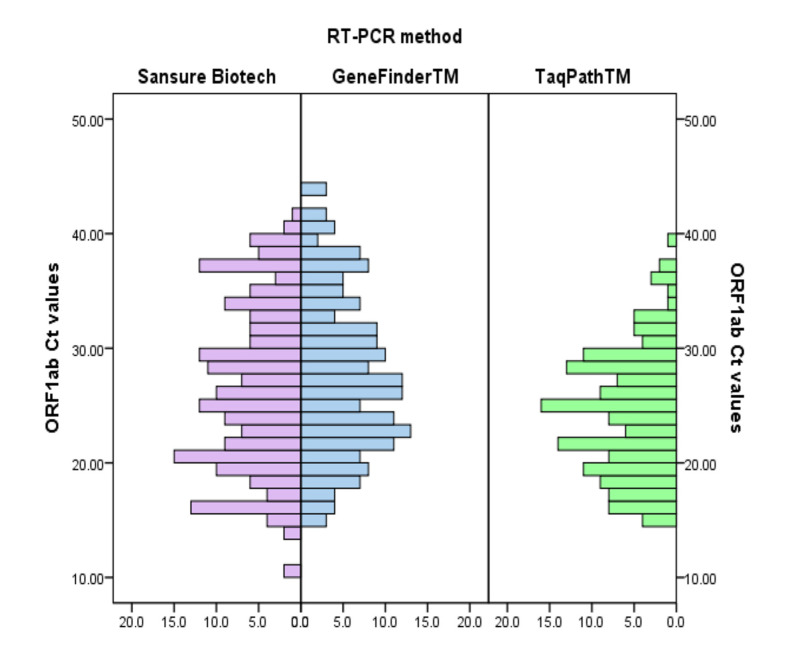
Distribution of Ct values that refer to amplification of ORF1ab gene according to the applied method.

**Figure 2:**
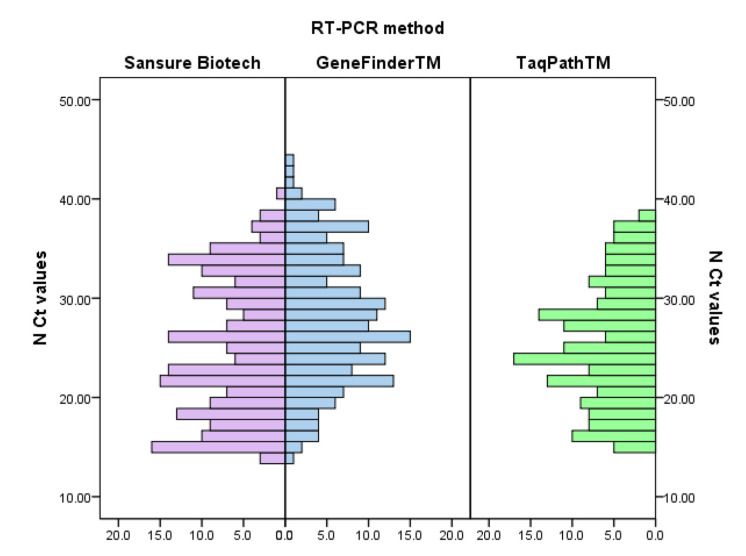
Distribution of Ct values for N gene according to the applied method.

The distribution of Ct values for the ORF1ab and the N genes according to the Sansure Biotech, GeneFinder^TM^, and TaqPath^TM^ methods are presented in Figure 1 and Figure 2.

## 4. Discussion

In this study, we provide a comparison of three available COVID-19 RT-qPCR kits from different manufacturers using patients specimens collected from the Serbian SARS-CoV-2 outbreak collected during March 2021. We were able to perform several observations, including a comparison of overall positivity, comparison of average Ct values that refer to amplification of single genes, the associations between Ct values obtained by different RT-qPCR kits and diagnostic accuracy of kits with sensitivity and specificity.

Although the number of positive samples differed among RT-qPCR kits, our findings showed that all three RT-qPCR methods have high diagnostic accuracy for SARS-CoV-2 infected patients with great inter-rater reliability. Some of these three kits have been previously analyzed also with a high agreement found after comparison between different commercial assays [10,12,13,14]. 

According to the divergence in the number of positive samples, there were 13 more after the Sansure Biotech RT-qPCR testing compared to the GeneFinder^TM^ or 28 compared to the TaqPath^TM^. Further analysis showed that the average Ct values that refer to amplification of the ORF1ab gene did not differ between the previously mentioned 13 samples and the rest of the positive samples obtained by the Sansure Biotech test. On the other hand, the average Ct values that refer to amplification of the N gene significantly differed between the same 13 samples and the rest of the positive samples obtained by the Sansure Biotech test. More precisely, the average Ct values that refer to amplification of N gene in 13 samples (that were interpreted as positive only according to the Sansure Biotech) were 36.02 ± 1.49, and in the rest of 194 positive samples (according to the Sansure Biotech) were 24.52 ± 6.62. This notable difference could not be properly interpreted without a reference method. However, Ct values that refer to the amplification of the N gene clearly show extreme values on its histogram and suggest possible false-positive results, especially since the other two tests from this study on the mentioned samples showed the absence of amplification of the N gene target.

Literature data theorizes that the potentially greatest sensitivity of the N target gene for SARS-CoV-2 detection is due to a higher abundance of sub-genomic N gene messenger RNAs in comparison to other targets [15]. Moreover, an increased detection window for the N gene compared to other target genes is also mentioned [13]. In addition to the data from our research, those theories are supported in a previously published study where it was strongly suggested that inclusion of the N gene target may improve sensitivity of SARS-CoV-2 detection [13]. This could be an explanation for the lower frequency of positive results of the samples when tested by the TaqPath^TM^ assay.

It is known that the Ct values indicate the level of virus concentration if the sample is taken correctly. Moreover, high Ct values may indicate false positivity and are often found in those patients who are no longer infectious [16,17]. Comparing the obtained Ct values of each of three tests from this study, we could conclude that there was a strong positive significant association between Ct values. However, the GeneFinder^TM^ assay showed the lowest efficiency to amplify the target gene since its average Ct values were significantly higher than in the other two RT-qPCR assays. 

During the global outbreak scenarios, the core of medical decision-making relies on accurate, rapid, and reliable laboratory results in both the inpatient and the outpatient settings. It directs the clinical management, introduction of prevention, or isolation measures, contact tracing, etc. After a year of developing and using different nucleic acid amplification assays, it is extremely important to identify and exclude those with low sensitivity and specificity firstly, to avoid reporting the false-positive results and unnecessary burdening of the health system with false-positive patients, and secondly, to prevent false-negative patients from further exposure and transmission of infection. All three tests from this study showed good sensitivity, with the highest one when using The Sansure Biotech, which ensures the recognition of true SARS-CoV-2 positive patients. However, the test specificity or ability to recognize true SARS-CoV-2 negative patients was not equal among all three assays. Therefore, the combination of good sensitivity and specificity is the most important feature. On the one hand, COVID-19 requires diagnostic tests with high sensitivity in life-threatening conditions, and on the other, high specificity is required because tests are in mass use. According to our study, a RT-qPCR kit with the best combination of sensitivity and specificity is the Sansure Biotech. As it is known that the likelihood ratio for a positive test (LR+) shows the enlargement of the estimated probability of being infected if the evaluated test was positive, and that tests with LR+ values over 10 indicate large and conclusive probability, it could be concluded that Sansure Biotech is a better predictor of COVID-19 sickness if this test is positive than GeneFinder^TM^ and that GeneFinder^TM^ is a better predictor of COVID-19 sickness if this test is positive than TaqPath^TM^. 

On 14 December 2020, British authorities announced that a new SARS-CoV-2 variant had been identified through viral genomic sequencing [18]. Soon after, in December 2020 and January 2021, national authorities in South Africa and Japan announced the detection of other new variants of SARS-CoV-2—B.1.351 and P.1, respectively [19,20]. More variants were reported during the first months of 2021, but only some of them were defined as variants of concern (VOC). All of these variants have characteristic mutations, the most significant of which are located in the gene encoding the spike (S) protein [21,22]. For example, the so-called “UK variant” B.1.1.7 is characterized by 23 mutations affecting ORF1ab, S, ORF8, and N [23]. The important consequence that refers to changes in S protein, as a target gene in RT-qPCR assays, includes so-called S-gene target failure or drop out. This target failure is not exclusive to any specific variant and it could be registered in both VOCs and non-VOCs. Even prior to the emergence of the B.1.1.7 in the United Kingdom, 1–5% of sequenced samples already had the deletion/target failure [24].

One of the important observations in this study is the absence of S gene detection using the TaqPath^TM^ kit. Since at the beginning of January 2021, during routine diagnosis with the same kit, we did not notice similar gene target failure, it could be interpreted as a consequence of some newly formed mutations, most likely deletion at nt207-212 in the respective gene, which is the characteristic of B.1.1.7. This was assumed according to unpublished but official publicly stated data which were related to the molecular epidemiology of SARS-CoV-2 in Serbia. Based on these data, the first B.1.1.7 isolate in Serbia was reported in the last week of January 2021, and during March, more than 80% of isolates belonged to B.1.1.7. During the period of sample collection for this study, the UK variant dominated in Serbian isolates; thus, our interpretation seems very plausible. In support of this interpretation, it has already been suggested that a negative or significantly weaker positive S-gene result with positive results for the other targets, in some multiplex RT-qPCR assays could serve as an indicator or screening method of potential circulation of the B.1.1.7 variant [24]. In order to investigate the precise reason for drop out, sequencing of all S-gene target failures is recommended. Interestingly, the S-gene target failure does not occur for B.1.351 and most probably not for lineage P.1 [24].

There are some study limitations to be mentioned. First, none of the tests that were examined in this study represent the gold standard. Second, to minimize the factors that could influence the final results of the testing, RNA extraction was performed under the same conditions regardless of RT-qPCR method which followed extraction. However, the Sansure Biotech kit includes its own lysis-based sample preparation reagent, which is also used in routine molecular diagnostics. It is less effective in the isolation of RNA, but is simpler and faster to use in situations of high laboratory load with samples for analysis.

## 5. Conclusions

In conclusion, the results from this study show high similarity in the analytical sensitivities for SARS-CoV-2 detection, which indicates that diagnostic accuracy of assays Sansure Biotech, GeneFinder^TM^ and TaqPath^TM^ are comparable. However, the Sansure Biotech assay showed a bit better diagnostic performance than other RT-qPCR methods. Moreover, the results provide an assessment of the tests performance characteristics at a time of advanced pandemic and increasingly frequent virus changes. Because of the crucial role that laboratory testing plays in surveillance to guide public health response and the lack of standardized diagnostic setup worldwide, it remains vital to maintain regular monitoring assays performance. Our findings suggest that the imperative for improvement should address the determination of cut-off Ct values and rapid modification of the primer sets along with the appearance of new variants.

## Figures and Tables

**Table 1:** Overview of molecular kits used in this study and rules of interpretation.

Assay	Target Genes	Internal Control	RNA (Template) Volume (μL)	Analytical Sensitivity (LOD-Limit of Detection)	Interpretation of Positive Results				
GeneFinder COVID-19 Plus Real*Amp* Kit (OSANG Healthcare Co., Anyang, Korea)	ORF1ab (RdRp E N	RNase P	5	500 copies per mL	ORF1ab (RdRp gene) Sigmoidal amplification curve and Ct ≤ 40	N gene Sigmoidal amplification curve and Ct ≤ 40		E gene Sigmoidal amplification curve and Ct ≤ 40	
					+	+		+	Positive
					If only one or both targets are positive			+/−	Positive
					-	-		+	“Presumptive positive” repeat the test. If the repeated result is the same report positive
Sansure Biotech (Sansure Biotech Inc., Changsha, China)	ORF1 ab N	RNase P	20	200 copies per mL	ORF1ab Sigmoidal amplification curve and Ct ≤ 40		N gene Sigmoidal amplification curve and Ct ≤ 40		
					+		+		Positive
					+		−		Positive
					−		+		Positive
TaqPath COVID-19 CE-IVD RT-PCR Kit (Thermo Fisher Scientific, Waltham, Massachusetts, USA)	ORF1 ab N S	MS2 Phage	10	10 genomic copies equivalents	Sigmoidal amplification curve and Ct ≤ 37 Two or more SARS-CoV-2 targets positive or If only one targeted gene is positive, repeat the test one more time, and if the result is the same, report positive				Positive

**Table 2:** Results and comparison of three different RT-qPCR methods tested on 354 specimens.

RT-qPCR Method	Target Gene	Positive, *n* (%)	Negative, *n* (%)
**Sansure Biotech**	Only ORF1ab	4 (1.1)	350 (98.9)
	Only N	12 (3.4)	342 (96.6)
	Both ORF1ab and N	190 (53.7)	164 (46.3)
**Sansure Biotech final result**		206 (58.2)	148 (41.8)
**GeneFinder^TM^**	Only ORF1ab	6 (1.7)	348 (98.3)
	Only N	11 (3.1)	343 (96.9)
	Only E	176 (49.7)	178 (50.3)
	Both ORF1ab and N	176 (49.7)	178 (50.3)
**GeneFinder^TM^ final result**		193 (54.5)	161 (45.5)
**TaqPath^TM^**	Only ORF1ab	0	354
	Only S	0	354
	Only N	24 (6.8)	330 (93.2)
	Both ORF1ab and S	0	354
	Both ORF1ab and N	132 (37.3)	222 (62.7)
	Both S and N	0	354 (100)
	ORF1ab and S and N	22 (6.2)	332 (93.8)
**TaqPath^TM^ final result**		178 (50.3)	176 (49.7)

**Table 3:** Ct values and comparison of three different RT-qPCR methods.

Gene	RT-qPCR Method Ct Value, Mean ± sd			*p* *			
	Sansure Biotech	GeneFinder^TM^	TaqPath^TM^		Sansure Biotech vs. GeneFinder^TM^	Sansure Biotech vs. TaqPath^TM^	GeneFinder^TM^ vs. TaqPath^TM^
**ORF1ab**	26.56 ± 7.26	27.80 ± 7.00	24.40 ± 5.51	<0.001	0.171	**0.008**	**<0.001**
**N**	25.03 ± 6.90	28.00 ± 6.69	25.46 ± 6.20	<0.001	**<0.001**	0.797	**0.001**
**E**	/	25.61 ± 6.01	/	NA	NA	NA	NA
**S**	/	/	27.03 ± 5.30	NA	NA	NA	NA

* for the level of significance of 0.05 according to One-Way ANOVA with Tukey posthoc testing.

**Table 4:** Correlation matrix for the association between Ct values obtained by the three RT-qPCR methods.

RT-qPCR Method	ORF1ab Gene			N Gene		
	SansureBiotech	GeneFinder^TM^	TaqPath^TM^	Sansure Biotech	GeneFinder^TM^	TaqPath^TM^
**Sansure Biotech**	1	r = 0.939 *p* < 0.001	r = 0.984 *p* < 0.001	1	r = 0.958 *p* < 0.001	r = 0.984 *p* < 0.001
**GeneFinder^TM^**		1	r = 0.938 *p* < 0.001		1	r = 0.949 *p* < 0.001
**TaqPath^TM^**			1			1

**Table 5:** Diagnostic accuracy of three RT-qPCR methods.

RT-qPCR Method	Measure of Diagnostic Accuracy with Its 95% CI						
	Sn (%)	Sp (%)	Overall Accuracy (%)	LR+	LR−	PPV (%)	NPV (%)
**Sansure Biotech vs. GeneFinder^TM^**	99.5 (98.5–1.00)	91.3 (87.0–95.7)	95.8 (93.7–97.9)	11.440 (6.94–18.87)	0.006 (0.001–0.040)	93.2	99.3
**GeneFinder^TM^ vs. TaqPath^TM^**	99.4 (98.3–1.00)	90.9 (86.7–95.2)	95.2 (93.0–97.4)	10.94 (6.86–17.45)	0.006 (0.001–0.044)	91.7	99.4
**Sansure Biotech vs. TaqPath^TM^**	99.4 (98.3–1.00)	83.5 (78.0–89.0)	91.5 (88.6–94.4)	6.03 (4.33–8.42)	0.01 (0.001–0.05)	85.9	99.3

## Data Availability

The data presented in this study are available on request from the corresponding author. The data are not publicly available due to institutional COVID-19 regulations.
